# The relationship between native T1 and mortality in patients requiring maintenance haemodialysis, using cardiac magnetic resonance imaging

**DOI:** 10.1016/j.jocmr.2025.101978

**Published:** 2025-10-24

**Authors:** Sherna F Adenwalla, Rachael E Stannard, Alastair J Rankin, Daniel S March, Jennifer S Lees, Gaurav S Gulsin, Gerry P McCann, James O Burton, Patrick B Mark, Matthew PM Graham-Brown

**Affiliations:** a Department of Cardiovascular Sciences, University of Leicester, UK; b NIHR Leicester Biomedical Research Centre, University Hospitals of Leicester NHS Trust, Leicester, UK; c Department of Health Sciences, University of Leicester, Leicester, UK; d School of Cardiovascular and Metabolic Health, University of Glasgow, 126 University Place, Glasgow, UK; e The Glasgow Renal & Transplant Unit, Queen Elizabeth University Hospital, 1345 Govan Road, Glasgow, UK; f National Centre for Sport and Exercise Medicine, School of Sport, Exercise and Health, Loughborough University, UK

**Keywords:** Haemodialysis, Cardiac MRI, Mortality, Global native T1, Cardiovascular, End-stage kidney disease

## Abstract

**Background:**

In people with kidney failure requiring haemodialysis, sudden cardiac death and arrhythmia are prevalent causes of mortality, driven by left ventricular (LV) hypertrophy and myocardial fibrosis. Native T1, a non-contrast magnetic resonance imaging (MRI) technique, is thought to represent myocardial fibrosis in this population. We hypothesised that MRI measures of cardiac structure and function would associate with mortality.

**Methods:**

A post-hoc, longitudinal analysis of data from the CYCLE-HD (ISRCTN11299707) and TICKER (NCT03704701) studies examined associations between native T1, LV mass index, LV ejection fraction (LVEF), and mortality over five years in people requiring haemodialysis. Cox proportional hazard models were adjusted for age, sex, diabetes, time on dialysis, and subsequent transplant.

**Results:**

Among 156 participants (mean age 60, 71% [111/156] male), 71 died over five years. In adjusted models increases in T1, LV mass index, and decreases in LV ejection fraction independently associated with mortality. A 30 ms increase in native T1 conferred a 27% increased risk of death. A 1 g/m^2^ increase in LV mass index increased mortality risk by 2%. Each 1% increase in LVEF reduced mortality risk by 3%. In an exploratoryadjusted model that included native T1, LVMi and LVEF, only LVMi remained an independent determinant of mortality.

**Conclusion:**

Native T1, LV mass index, and LVEF were significant determinants of mortality in people requiring haemodialysis. T1 shows promise as a novel biomarker of myocardial fibrosis in this population. Associations between changes in T1 and mortality, and consideration of implementing findings into clinical practice needs research.

## Graphical abstract

**Figure:**
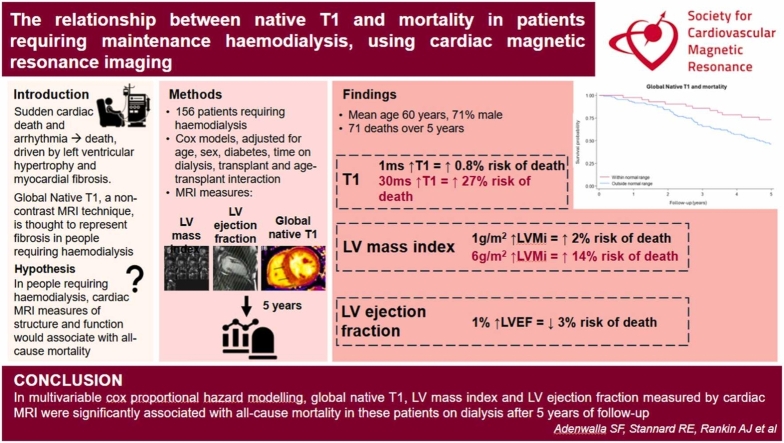

## Introduction

People with kidney failure requiring haemodialysis have significantly elevated risk of death from cardiovascular disease than the general population [1]. The most frequent modes of death are sudden cardiac death and fatal arrythmia, rather than classical atheromatous ischaemic heart disease and myocardial infarction [2]. Indeed, much of this excess cardiovascular risk is linked to and driven by pathological changes in left ventricular (LV) structure and function [3]. These changes include LV hypertrophy, LV cavity dilatation and related LV systolic and diastolic dysfunction [4]. Left ventricular hypertrophy has been suggested as a determinant of outcomes for these patients [5], [6], [7], although the strength of its relationship to mortality has been questioned [8]. The pathogenesis of LV hypertrophy is complex, but relate to changes in both preload and afterload, systemic hypertension and demand ischaemia resulting from the processes of dialysis themselves [9], [10]. These same processes drive the development of non-coronary artery disease related interstitial myocardial fibrosis [11]. Left ventricular hypertrophy and fibrosis contribute towards the heart failure with preserved ejection fraction phenotype, which accounts for approximately half of heart failure syndromes in patients with kidney failure and towards the burden of multimorbidity in this population [12]. Indeed, histopathological studies suggest extent of interstitial myocardial fibrosis is the strongest determinant of cardiovascular outcome [13], [14].

Assessment of myocardial fibrosis with gadolinium enhanced cardiac MRI is an established clinical and research tool for many disease groups and is highly predictive of outcomes [15], [16]. Gadolinium based contrast agents are, however, relatively contraindicated in people with kidney failure requiring haemodialysis due to the association of the use of ‘linear’ gadolinium-based agents and the debilitating condition nephrogenic systemic fibrosis (NSF) [17]. Whilst the risk of NSF is reduced with newer gadolinium agents, caution is still recommended [18]. Native T1 mapping is a non-contrast cardiac MRI technique that has emerged as a potential measure of myocardial fibrosis in people requiring dialysis [19], [20], [21], [22], [23]. T1 relaxation represents longitudinal recovery time of hydrogen atoms following excitation. Increased native T1 time has been shown to be a surrogate marker of myocardial fibrosis in disease states [24], [25], [26], [27], and histological studies in people with kidney failure requiring dialysis are awaited (NCT03586518). A small study has reported elevated native T1 times are predictive of major adverse cardiovascular events (MACE) in people requiring haemodialysis, but was limited by both patient numbers and follow-up [28]. Improved understanding of the relationship between native T1 and mortality for people with kidney failure requiring replacement therapy is needed to define the utility of native T1 as a tool in research studies and possibly as a clinical tool to improve risk stratification.

In this study we assessed the relationships between native T1, LV hypertrophy, and LV ejection fraction (LVEF) and long-term survival in people with kidney failure requiring haemodialysis recruited to clinical studies at two UK centers.

## Methods

Data for this study are taken from the baseline cardiovascular MRI (CMR) and associated clinical data from patients recruited to two separate studies at two UK centers. The CYCLE-HD study and TICKER study recruited people with kidney failure requiring maintenance haemodialysis [29], [30]. All patients underwent comprehensive phenotyping at baseline with multi-parametric CMR on a 3 T platform. Both studies received ethical approval (CYCLE-HD, REC ref: 14/EM/1190; TICKER, REC ref: 18/WS0138) and all patients gave written informed consent. Inclusion and exclusion criteria are as previously published, and included patients aged above 18 years (TICKER inclusion criteria aged over 40 years) and able to provide written informed consent. In both studies patients needed to have been on a maintenance dialysis programme for over 3 months.

### Demographic data

Demographic information including age, sex, ethnicity, cause of kidney failure, past medical history and comorbidities (extracted from coded electronic records or self-report), haematological and biochemical data were recorded at the time of baseline CMR scan. Total time on dialysis was calculated in months.

### CMR acquisition

Patients enrolled in the CYCLE-HD study were scanned at center 1 on a 3 T platform (Skyra, Siemens Healthineers, Erlangen, Germany) with an 18-channel phased array receiver coil. Patients enrolled in the TICKER study were scanned at center 2 on a Siemens Prisma 3 T scanner (Siemens Healthineers) with an 18-channel body array used anteriorly with a 32-channel spine array for posterior acquisition.

Different CMR protocols were used at centers 1 and 2 due to local expertise in gaining the highest quality images with the fewest number of artifacts.

#### CMR protocol center 1

CMR imaging was performed on a non-dialysis day, not after the ‘long-gap’. Electrographic (ECG) gated, breath-held steady-state free procession long-axis cine images in 2, 3 and 4 chamber views were acquired. Short-axis cine images covering the entire left ventricle were taken at 8 mm slice thickness, 2 mm gap, field of view 300 ×400 mm, matrix 208 ×256, repetition time 2.9 ms, echo time 1.2 ms, flip angle 64–790, temporal resolution <50 ms, with 30 phases per cardiac cycle, in-plane image resolution 1.1 ×1.5 mm to 1.3 ×1.7 mm. Short-axis mid-ventricular and basal native T1 maps were acquired using the modified look-locker inversion recovery (MOLLI) sequence. Images were acquired using free-breathing with motion correction (MOCO), ECG-gated single-shot MOLLI sequence [23], with 3(3)3(3)5 sampling pattern and the following typical parameters: slice thickness 8.0 mm, field of view 300 × 400 mm, flip angle 50°, minimum TI 120 ms, inversion-time increment 80 ms as previously described [22].

#### CMR protocol center 2

CMR imaging was performed on a non-dialysis day, not after the ‘long gap’ (dialysis patients typically dialyse three times per week, so one dialysis session occurs after a 2-day gap and is described as the ‘long gap’. Patients at this point are more likely to be fluid overloaded). Image acquisition was ECG-gated [23]. Electrographic gated, breath-held steady-state free procession long-axis cine images in 2, 3 and 4 chamber views were acquired. Short axis cine images covering the entire left ventricle were taken at 7 mm slice thickness, 3 mm gap, field of view 340 ×286 mm, repetition time 41.4 ms, echo time 1.51 ms, flip angle 50°, temporal resolution <50 ms, with 25 phases per cardiac cycle, voxel size 1.33 ×1.33 ×7 mm. Where participants were unable to breath-hold or had cardiac arrhythmia, compressed sensing (CS cardiac Cine, Siemens Healthineers) was used to allow real-time acquisition. Native T1 maps were acquired in basal and mid ventricular short axis views using the same MOLLI sequence as at center 1. Typical acquisition parameters were: slice thickness 6.0 mm, field of view 340 ×272 mm, flip angle 35°, minimum T1 100 ms, inversion-time increment 80 ms, repetition time 272 ms, bandwidth 1085 Hertz/pixel [30].

### CMR analysis

Analyses were completed separately at center 1 and center 2. The inter-center reproducibility of measures of left ventricular structure and function and native T1 between centers 1 and 2 are excellent as previously reported [31]. All CMR analyses were completed on anonymised scans using the CVI42 software package (Circle Cardiovascular Imaging, Calgary, Alberta, Canada) by experienced readers independent of all other study data.

Left ventricular mass (LVM) and volumes were calculated by manually drawing endocardial and epicardial contours in end-diastole and end-systole on LV short axis cine images, papillary muscles in LV volume calculations, as previously described and conforming to international guidelines with indexing of mass and volumes to body surface area [32]. For native T1 times epi- and endocardial contours were drawn on basal and mid ventricular native T1 maps and the average of the mid and basal native T1 times were taken as global native T1 as previously described [31]. Areas of obvious artifact were excluded from analysis.

### Follow up

Follow-up data (mortality and kidney transplantation since study enrolment) were collected from electronic patient records at both centers.

### Statistical analyses

Statistical analysis was undertaken using Stata/BE version 18 (StataCorp, College Station, TX, US). Normally distributed data are expressed as mean ± standard deviation and non-normally distributed data are expressed as median (interquartile range). Cox proportional hazard models were used for time to event analysis. Three separate multivariable models were used, with each MRI measure (Global Native T1, LV mass index, and LVEF) and pre-determined covariates (age, sex, diabetes, time on dialysis, receiving a transplant since trial enrolment). Receiving a transplant was treated as a time-varying covariate. Interaction effects between age and transplant, age and T1, and diabetes and age were considered but not selected. The proportional hazards assumption was assessed for each covariate (continuous – Schoenfeld residual plots, categorical - "log-log" plots). There was no evidence of violation of the proportional hazards assumption for any CMR measure or covariate, as indicated by a smooth horizontal line. The functional form of continuous covariates and MRI measures was evaluated using martingale residual plots. Linear, log transform, quadratic polynomial, and spline forms (three degrees of freedom) were considered. The linear form was selected for each CMR measure and continuous covariates due to the absence of evidence for the need of a more complex form. The martingale residual plots were flat and smooth with no clear pattern. Where covariates exhibited small deviation from this horizontal line in the tails, the martingale residual plots were not improved by using log transform, quadratic polynomial, or spline terms. Models were compared using the Akaike information criterion (AIC) and likelihood ratio test and evaluation of confidence intervals. Follow-up was restricted to five years since trial enrolment to ensure equal follow-up of patients from both centres. Kaplan-Meir graphs were used to visually represent unadjusted survival data for the MRI measures.

In an exploratory analysis, native T1, LVMi and LVEF were all included in a model with the same pre-determined co-variates to assess for the independence of these variables. Further exploratory analyses assessed the relationships between native T1, LVMi and LVEF and cardiovascular mortality and the relationships between native T1, LVMI and LVEF and all-cause mortality in patients who were not transplanted and remained on haemodialysis in models with the same pre-determined co-variates.

## Results

Baseline patient demographics and CMR parameters are shown in Table 1. Of 156 patients included, the mean age was 60 (50, 69) years, 71% (111/156) were male and the median duration of dialysis was 1.4 (0.6, 3.6) years. Approximately 70% of the population had hypertension, and 40% had diabetes. After five years of follow up since baseline data collection, 53 patients on dialysis had received a renal transplant and 71 had died. Causes of death are shown in Table 2. Nine deaths occurred in patients who had received a kidney transplant and seven of these individuals had T1 values above the normal range. Sixty-two deaths occurred in patients who were not transplanted and remained on dialysis and 10 of these had T1 values in the normal range.

**Table 1:** Demographic and baseline data of the study population.

	Whole cohort (n=156)	Alive at follow-up (n=85)	Dead at follow-up (71)
Age (years)	60 (50, 69)	65 (58, 75)	53 (40, 51)
Male sex, n (%)	111 (71%)	68 (80%)	43 (51%)
Dialysis vintage (years)	1.4 (0.6, 3.6)	1.34 (0.6, 3.8)	1.5 (0.7, 3.4)
BMI (kg/m^2^)	27 (23, 31)	27 (23, 31)	27 (24, 32)
Pre-dialysis average SBP (mmHg)	146 (127,161)	148 (128, 166)	142 (125, 157)
Pre-dialysis average DBP (mmHg)	76 (64, 86)	75 (62, 88)	78 (69, 85)
Haemoglobin (g/L)	112.5 ± 14.0	111 ± 14	114 ± 14
Albumin (g/L)	37 (33,40)	36 (32, 39)	38 (34, 41)
Total cholesterol (mmol/L) (n=127)	3.8 (3.2, 4.7)	3.7 (3, 4.5)	4 (3.3, 4.9)
*Ethnicity*			
White, n (%)	80 (51%)	37 (44%)	43 (61%)
Mixed or multiple ethnic groups, n (%)	2 (1%)	2 (2%)	0 (0%)
Asian/Asian British, n (%)	55 (35%)	33 (39%)	22 (31%)
Black/Black British, n (%)	12 (8%)	7 (8%)	5 (7%)
Other, n (%)	7 (4%)	6 (7%)	1 (1%)
*Cause of renal disease*			
Diabetic kidney disease	33 (21%)	19 (22%)	14
Primary glomerulonephritis	22 (14%)	10 (12%)	12
Interstitial nephritis	5 (3%)	3 (4%)	2
Obstructive uropathy/chronic pyelonephritis	8 (5%)	3 (4%)	5
Renovascular disease	9 (6%)	5 (6%)	4
Polycystic kidney disease	8 (5%)	5 (6%)	3
Other	32 (21%)	17 (20%)	15
Unknown	39 (25%)	24 (28%)	17
*Comorbidities*			
Current smoker, n (%) (n=141)	21 (15%)	11	10
Hypertension, n (%)	110 (71%)	59	51
Ischaemic heart disease, n (%)	23 (15%)	16	7
Diabetes, n (%)	61 (39%)	41	20
*CMR parameters*			
Global Native T1 (ms) (n=150)	1271 ± 42	1261 ± 42	1281 ± 41
LV mass index (g/m^2^)	58.8 (48, 75)	54 (43, 70)	60 (43, 70)
LV ejection fraction (%)	55 (47, 61)	56 (50, 62)	53 (45, 60)
LV end diastolic volume (ml)	169 ± 55	166 ± 56	172 ± 55
LV end systolic volume (ml)	83 ± 39	75 ± 36	86 ± 41
LV stroke volume (ml)	88 ± 26	90 ± 27	86 ± 25

Normally distributed data presented as mean ± SD, non-normally distributed data presented as median (25th, 75th percentile). LV indices indexed to body surface area.

*BMI* body mass index, *CMR* cardiovascular magnetic resonance, *SBP* systolic blood pressure, *DBP* diastolic blood pressure, *LV* left ventricular

**Table 2:** Causes of death for the 71 patients who had died at follow-up.

Cause of death	Number (%)
Cardiovascular disease	26 (37%)
Infection (non-COVID−19)	22 (31%)
Infection (COVID−19)	8 (11%)
Malignancy	3 (4%)
Treatment withdrawal	4 (6%)
Cerebrovascular event	4 (6%)
Other	4 (6%)

### Univariate associations of CMR parameters and mortality

Results of the univariate Cox proportional hazard regressions are presented in Table 3. Global Native T1, LV mass index and LV ejection fraction were each significantly associated with survival time after five years of follow-up.

**Table 3:** Univariate and multivariable cox proportional hazard models for each CMR parameter and date of death after five years of follow-up.

	Univariate model	Multivariable model	
Variable	Hazard ratio (95% CI)	Hazard Ratio (95% CI)	p-value
Global Native T1 (ms) (n=150)	1.010 (1.004, 1.016)	1.008 (1.002, 1.014)	0.01
Received Transplant		0.518 (0.222, 1.208)	0.13
Age (years)		1.055 (1.031, 1.079)	<0.01
Female		0.983 (0.534, 1.811)	0.96
Diabetes		0.698 (0.429, 1.137)	0.15
Time on Dialysis (years)		0.902 (0.805, 1.010)	0.07

LV mass index (g/m^2^)	1.013 (1.002, 1.024)	1.022 (1.010, 1.035)	<0.01
Received Transplant		0.482 (0.220, 1.057)	0.68
Age (years)		1.062 (1.039, 1.085)	<0.01
Female		1.230 (0.692, 2.185)	0.48
Diabetes		0.676 (0.421, 1.085)	0.11
Time on Dialysis (years)		0.882 (0.783, 0.994)	0.04

LV ejection fraction (%)	0.960 (0.939, 0.981)	0.972 (0.950, 0.994)	0.01
Received Transplant		0.483 (0.219, 1.065)	0.07
Age (years)		1.050 (1.027, 1.073)	<0.01
Female		1.145 (0.643, 2.020)	0.65
Diabetes		0.698 (0.436, 1.116)	0.13
Time on Dialysis (years)		0.886 (0.787, 0.997)	0.04

The reference category for Received transplant is ‘no’ and Diabetes is ‘yes’.

*CI* confidence interval, *LV* left ventricular

### Multivariable associations of CMR parameters and mortality

All CMR parameters were each independently associated with survival time after adjustment with pre-determined variables (Table 3).

#### Global Native T1 and mortality

**Fig. 1:**
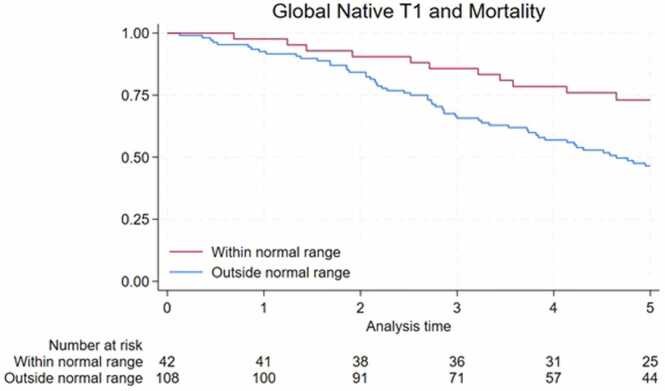
Kaplan-Meier survival estimate for patients with T1 values above and below the normal range over five years of follow-up. Normal range defined as upper limit of normal (1245 ms in center 1 and 1233 ms in center 2).

Assuming a clinically meaningful effect-size of 30 ms for people requiring dialysis [29], [33], a 30 ms increase in native T1 associated with a 27% increased risk of death. A Kaplan-Meier survival curve comparing survival of patients with T1 values above and below the normal range (upper limit of normal 1245 ms at center 1 and 1233 ms in center 2) is presented in Fig. 1.

#### LV mass index and mortality

A 1 g/m^2^ increase in LV mass index was associated with a 2% increase in the risk of death. Using a clinically meaningful effect-size of 6 g/m^2^ [34], a 6 g/m^2^ increase in LV mass index would be associated with a 14% increase in the risk of death. Kaplan-Meier survival curves comparing survival of patients with LV mass index values within and outside the normal range are presented in Supplemental materials, Fig. S1. Whilst men with increased LV mass index were at greater risk of death than those with normal LV mass index, this difference was not apparent in female participants in Kaplan-Meier survival analyses, although the sample size for female participants was small.

#### LV ejection fraction and mortality

A 1% increase in LV ejection fraction was associated with a 3% reduction in risk of death. Kaplan-Meier survival curves comparing survival of patients with LV ejection fraction values within and outside the normal range are presented in Supplemental materials, Fig. S2.

#### Exploratory analysis of relationship between CMR variables and mortality in patients who remained on haemodialysis

Fifty three patients were transplanted during follow-up. In an exploratory analysis the relationship between CMR variables and mortality was assessed in the 97 patients who remained on dialysis. In the multivariable models, hazard ratios were similar, but with wider confidence intervals due to the reduction in power and native T1 and LVEF just lost significance in the models in this exploratory analysis (supplemental table S1).

#### Exploratory analysis of relationships between CMR variables and cardiovascular mortality

Twenty six patients in the cohort had a cardiovascular cause of death. In an exploratory analysis the relationships between CMR variables and cardiovascular mortality were largely unchanged in adjusted and unadjusted models, with wider confidence intervals due to the smaller number of end points (supplemental table S2).

#### Exploratory analysis of relationships between native T1, LVMi and LVEF and mortality

In an exploratory analysis, LVMi remained an independent determinant of mortality (hazard ratio: 1.017 (1.001, 10.32, p=0.04) in a model that included native T1, LVEF and the same pre-determined covariates. Native T1 just missed the threshold to be considered an independent determinant of mortality in this model (hazard ratio: 1.006 (1.000, 1.012, p=0.07) (Table 4).

**Table 4:** Multivariable cox proportional hazard model including the CMR parameters native T1, LV mass index and LV ejection fraction together in addition to pre-determined co-variates and date of death after 5-years of follow-up.

Variable	Hazard Ratio (95% CI)	**p-value**
Global Native T1 (ms)	1.006 (1.000, 1.012)	0.07
LV mass index (g/m^2^)	1.017 (1.002, 1.033)	0.03
LV ejection fraction (%)	0.992 (0.966, 1.020)	0.57
Received Transplant	0.515 (0.221, 1.201)	0.013
Age (years)	1.060 (1.036, 1.085)	<0.01
Female	1.147 (0.622, 2.115)	0.66
Diabetes	0.704 (0.430, 1.151)	0.16
Time on Dialysis (years)	0.893 (0.792, 1.007)	0.07

The reference category for Received transplant is ‘no’ and Diabetes is ‘yes’.

*CI* confidence interval, *LV* left ventricular

### Sensitivity analyses

The hazard ratio for each CMR measure was estimated several times under different modelling assumptions (non-proportional hazards, alternate functional form for continuous covariates and including more interaction effects). The hazard ratio remained largely invariant under these different assumptions. These data are included as Supplemental materials, Fig. S3. In post-hoc analysis, separate confidence intervals were obtained for each covariate via bootstrapping with 1000 repetitions to assess model stability. These can be found in Supplemental materials, Tables S3-S5.

## Discussion

This study examined associations between native T1 times, LVMi and LVEF and mortality in a cohort of patients with kidney failure requiring haemodialysis. After adjustment for important clinical variables, we found a linear relationship between increased native T1 and all-cause mortality. Patients with an elevated native T1 were more likely to die within 5 years than those with a normal native T1. As previously described, there is a graded relationship between both LVMi and LVEF , and all-cause mortality. Our findings are consistent with the published literature in people requiring haemodialysis [35], [36]. In an exploratory model, only LVMi remained an independent determinant model when LVMi, native T1 and LVEF were included together, with native T1 just missing the threshold to be considered an independent determinant.

Native T1 times are known to be elevated in people requiring haemodialysis and in people with non-dialysis chronic kidney disease (CKD) [21], [22], [23], [37]. In this population, T1 associates with measures of acute myocardial injury [23], and subclinical systolic dysfunction [22]. We do not yet have histological confirmation that T1 is a direct surrogate for myocardial fibrosis, and elevated times may reflect inflammation or myocardial oedema [38]. However, changes in T1 before and after dialysis are small [30], and there are no significant differences between native T2 times (measure of myocardial oedema) in patients requiring dialysis and control subjects [39]. Native T1 has been shown to be highly reproducible in this population [31] and is modifiable in interventional studies, including exercise and novel dialysis regimens [29], [33]. Moreover, although native T1 times do not decrease immediately following kidney transplantation (two months post) [40], longer follow-up (six months post) show that native T1 does reduce following kidney transplantation [41], in keeping with the reduction in fibrosis and LV pathology that is thought to occur following transplantation [13].

Elevated native T1 times associate with adverse patient outcomes in people with amyloidosis [42], non-ischaemic cardiomyopathy [43], dilated cardiomyopathy [44], and predicted incident cardiovascular events and all-cause mortality in the Multi-Ethnic Study of Atherosclerosis [45]. Native T1 was shown to be a determinant of major adverse cardiovascular events (including all-cause mortality) in people with CKD and aortic stenosis [46]. In the study by Ramchand et al., 6/117 patients included were on dialysis, with a median eGFR in the cohort of around 30 ml/min/m^2^. Whilst our study included a very different population, the findings described by Ramchand et al. are in keeping with the findings of this study and in a population where there is histological confirmation that native T1 times correlate with increased levels of interstitial fibrosis [24]. Furthermore, native T1 is known to improve in the 12 months following valve replacement surgery in people with severe aortic stenosis and those with greater improvements are more likely to see improvements in LV function and prognosis [47]. Our findings also support the preliminary findings of Qin et al. [28], who showed that the risk of MACE was greater in people requiring dialysis with an elevated T1. The event rate for this study was low and in small numbers, but it is encouraging that results are consistent. Our findings describe a graded, linear relationship between T1 and mortality risk, not just outcome differences between patients with normal and elevated T1 values. This makes biological sense as extent of myocardial fibrosis is closely linked to outcome. The data we present are an important addition, given the larger numbers, longer follow-up, greater event rate and ability to adjust for a greater number of covariates, but larger prospective studies are now warranted.

This study confirms that changes in LV mass index and LV ejection fraction associate with all-cause mortality. Historically, LV mass has been viewed as a biomarker of cardiovascular risk and mortality in people on haemodialysis with many concluding that LV mass could be used to risk stratify patients and optimise therapies [7]. These predictions have not translated into routine clinical care and part of the reasons for this is perhaps that changes in LV mass do not uniformly associate with changes in patient outcomes when CMR and echocardiographic data are included altogether [8]. Indeed, many of the studies that show strong relationships between LV mass (and changes in LV mass) and outcomes were conducted with echocardiography which are prone to overestimate LV mass in states of volume overload [48]. Left ventricular mass index remained an independent determinant of mortality when included in a model with native T1, LVEF and other co-variates, in keeping with previous studies that have shown LVMi to be a reliable determinant of mortality. Native T1 missed the threshold to be considered an independent determinant of mortality when included with LVMi, but only by a very small amount. This was an exploratory, underpowered analysis, and should be viewed as hypothesis generating. Further work is warranted in larger prospective studies to assess whether native T1 is an independent biomarker of risk and patient outcomes that adds additional information to measuring LVMi. Myocardial fibrosis is an early marker of cardiovascular disease and may be detectable with native T1 before overt changes in LV structure and function. Moreover, histopathological studies confirm that degree of non-coronary artery disease related interstitial myocardial fibrosis is a stronger determinant of cardiovascular outcome in patients with advanced CKD and on dialysis than LV mass [13], [14]. At present, it is difficult to see how native T1 assessment could be integrated into the routine clinical care of people requiring haemodialysis given the limitations in access to CMR scanning and T1 mapping. Unless there are increases in capacity and skill, native T1 will remain a potentially useful tool in research studies and *if* validation studies confirm native T1 as a clinically useful tool for risk stratification then consideration must be given to the availability of the technique to support implementation of research findings in clinical care. Furthermore, there are many factors that influence native T1 relaxation time, including field-strength, scanner model and sequence. The future clinical utility of native T1 in this population would require these issues to be suitably addressed. The observation that there were differential outcomes for males and females with elevated or normal LV mass index must be interpreted cautiously as the absolute numbers of females and the event rates in females in the sample are too small to draw meaningful conclusions.

## Limitations

The major limitation of this study is its post-hoc design and relatively small number of participants. This limits the numbers of covariates that can be included in models and these findings should all be validated in larger, prospective studies that also consider the relative contributions of different (CMR) measures of cardiovascular disease within the same models. As data are from one point in time, the competing effects of changes in other treatments over time (e.g. angiotensin converting enzyme inhibitors/angiotensin receptor blockers, haemoglobin levels or iron stores) cannot be accounted for. There were only 26 cardiovascular deaths in the cohort, limiting the extent to which we can comment on the relationship between CMR measures and cardiovascular death. However, in an exploratory analysis the relationship between all CMR measures and cardiovascular mortality was similar to overall mortality. Additionally, 53 participants received a kidney transplant and these patients had a lower mortality. Kidney transplantation was adjusted for as a confounder in cox models, but in an additional sub-group analysis the hazard ratios in multivariable models for the relationships between CMR variables and mortality were similar in a patients who did not receive a kidney transplant, accepting that in this sub-group native T1 and LVEF lost ‘significance’ in multi-variable models likely due to the reduced sample size and loss of power. In people requiring dialysis, volume status can always affect all measures of LV mass and volumes, as well as potentially small changes in native T1, but all patients in this study were scanned on their non-dialysis day at ‘dry’ ideal body weight, mitigating these effects. Furthermore, test-retest reproducibility of native T1 times in people requiring haemodialysis shows no relationship between changes in native T1 and changes in measures of volume status [31]. Whilst some interventional studies have shown a 30 ms change is achievable in native T1 times in people requiring haemodialysis, the minimal clinically important difference is not yet defined which is needed to fully understand the relevance of the associations we describe between T1 and mortality. Indeed, defining the minimally clinically important difference for native T1 is needed before clinical studies can be powered using this as a primary outcome measure in phase 2 mechanistic clinical trials.

## Conclusion

In this post-hoc, longitudinal and observational analysis, global native T1, LVMi and LVEF were significant determinants of all-cause mortality in people requiring haemodialysis after five years of follow-up. The association between T1 and mortality is novel. Further work is needed to validate these findings and establish the value of assessing native T1 in addition to other prognostically important measures of cardiovascular disease. Future studies should also consider whether findings could feasibly be implemented into clinical practice.

## Funding

This article presents independent research funded by the NIHR in the United Kingdom (grant reference number CS-2013–13-014; JOB) and supported by Kidney Research UK. JOB is funded (Senior Investigator Award) by the National Institute for Health and Care Research (NIHR). AJR is personally funded by a Clinical Academic Training Fellowship from The Chief Scientist Office (Scotland) (CAF/18/02). JSL is funded by a Wellcome Trust Early Career Award (301005/Z/23/Z). SFA, Academic Clinical Fellow (ACF-2021–11-002), is funded by NHS England (formerly Health Education England)/ National Institute for Health and Care Research (NIHR). The views expressed are those of the authors and not necessarily those of the National Health Service, the NIHR, or the Department of Health and Social Care. After the award of funding, the funder (NIHR) did not have any input into trial design, data collection, data analysis, data interpretation, in writing the report, or in deciding to submit for publication. This research has no relationship with industry.

## Declaration of Competing Interest

The authors declare the following financial interests/personal relationships which may be considered as potential competing interests. This article presents independent research funded by the NIHR in the United Kingdom (grant reference number CS-2013–13-014; JOB) and supported by Kidney Research UK. JOB is funded (Senior Investigator Award) by the National Institute for Health and Care Research (NIHR). Alastair J Rankin is personally funded by a Clinical Academic Training Fellowship from The Chief Scientist Office (Scotland) (CAF/18/02. If there are other authors, they declare that they have no known competing financial interests or personal relationships that could have appeared to influence the work reported in this paper.

## Data Availability

Data available on request. The statistical analysis code used for this manuscript is available to view: https://github.com/sfaden12/global-native-T1-and-mortality-in-patients-requiring-HD-/tree/main.

## Footnotes

Appendix A Supplemental data associated with this article can be found in the online version at doi:10.1016/j.jocmr.2025.101978.
