# Supplementary material for: The relationship between native T1 and mortality in patients requiring maintenance haemodialysis, using cardiac magnetic resonance imaging
Source: J Cardiovasc Magn Reson. 2025 Oct 24;27(2):101978. doi: 10.1016/j.jocmr.2025.101978 (PMC12744331; doi:10.1016/j.jocmr.2025.101978)
Supplement: Supplementary file 1 — Supplemental material [file mmc1.docx]

## Supplementary Material

### Figures

**
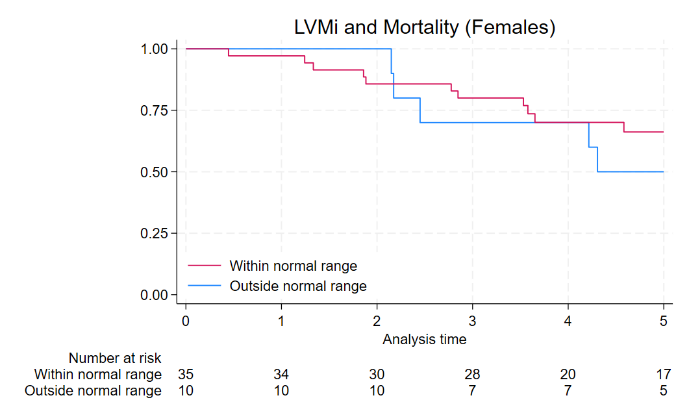

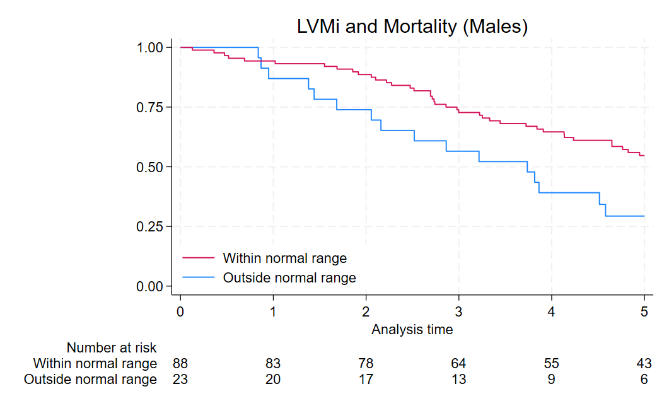
**

Figure S1: Kaplan-Meier survival estimate for patients with LV mass index within and outside the normal range over five years of follow-up. Normal range defined as 39-85 g/m2 for males and 30-68 g/m2 for females.

**
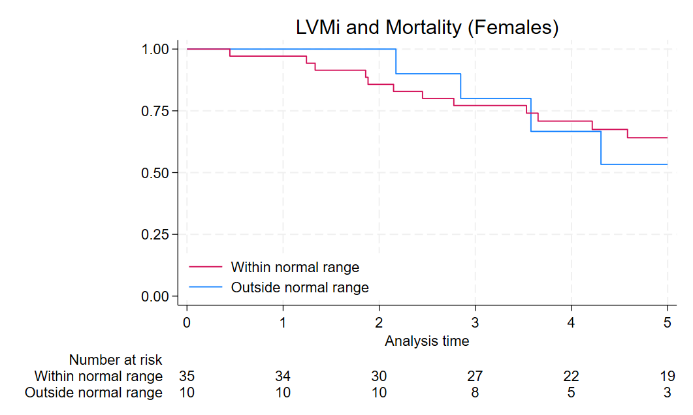

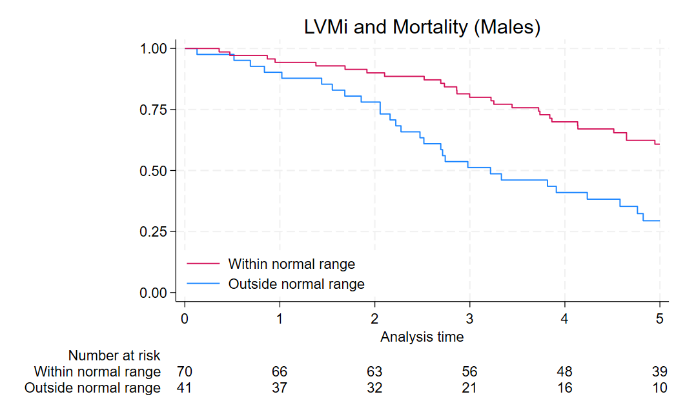
**

Figure S2: Kaplan-Meier survival estimate for patients with LV ejection fraction within and outside the normal range over five years of follow-up. Normal range defined as 49-79% for males and 52-79% for females.


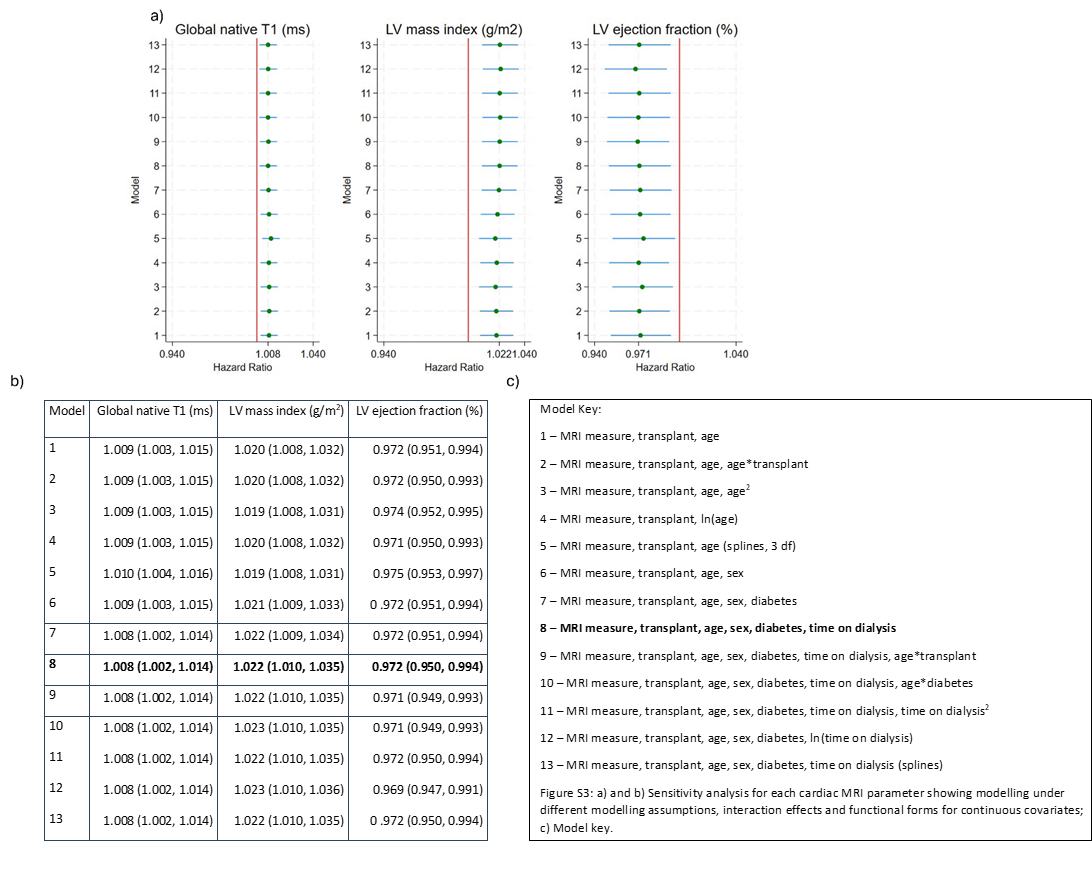


Figure S3: a) and b) Sensitivity analysis for each cardiac MRI parameter showing modelling under different modelling assumptions, interaction effects and functional forms for continuous covariates; c) Model key.

### Supplemental Tables

|  | **Multivariable model** | |
| --- | --- | --- |
| **Variable** | **Hazard Ratio (95% CI)** | **p-value** |
| **Global Native T1 (ms) (n=97)** | 1.005 (0.999, 1.012) | 0.11 |
| Age (years) | 1.033 (1.007, 1.060) | 0.01 |
| Female | 0.905 (0.462, 1.774) | 0.77 |
| Diabetes | 0.813 (0.487, 1.359) | 0.43 |
| Time on Dialysis (years) | 0.904 (0.799, 1.023) | 0.77 |
|  | | |
| **LV mass index (g/m^2^)** | 1.018 (1.005, 1.032) | 0.01 |
| Age (years) | 1.044 (1.019, 1.070) | <0.01 |
| Female | 1.096 (0.577, 2.083) | 0.78 |
| Diabetes | 0.736 (0.444, 1.219) | 0.23 |
| Time on Dialysis (years) | 0.878 (0.772, 1.000) | 0.05 |
|  | | |
| **LV ejection fraction (%)** | 0.980 (0.957, 1.004) | 0.10 |
| Age (years) | 1.032 (1.007, 1.057) | 0.01 |
| Female | 1.006 (0.529, 1.913) | 0.65 |
| Diabetes | 0.781 (0.475, 1.283) | 0.33 |
| Time on Dialysis (years) | 0.886 (0.780, 1.007) | 0.06 |

Supplemental table S1: Relationship between CMR variables and mortality in patients who did not receive a transplant and remained on dialysis.

|  | **Univariate model** | **Multivariable model** | |
| --- | --- | --- | --- |
| **Variable** | **Hazard ratio (95% CI)** | **Hazard Ratio (95% CI)** | **p-value** |
| **Global Native T1 (ms) (n=150)** | 1.015 (1.005, 1.025) | 1.013 (1.002, 1.024) | 0.02 |
| Received Transplant |  | 0.342 (0.073, 1.610) | 0.18 |
| Age (years) |  | 1.030 (0.990, 1.072) | 0.14 |
| Female |  | 0.856 (0.277, 2.643) | 0.79 |
| Diabetes |  | 0.412 (0.168, 1.008) | 0.05 |
| Time on Dialysis (years) |  | 0.962 (0.812, 1.139) | 0.65 |
|  | | | |
| **LV mass index (g/m^2^)** | 1.025 (1.007, 1.043) | 1.038 (1.015, 1.061) | <0.01 |
| Received Transplant |  | 0.292 (0.064, 1.331) | 0.11 |
| Age (years) |  | 1.053 (1.012, 1.096) | 0.01 |
| Female |  | 1.167 (0.377, 3.612) | 0.79 |
| Diabetes |  | 0.328 (0.134, 0.805) | 0.02 |
| Time on Dialysis (years) |  | 0.946 (0.774, 1.157) | 0.59 |
|  | | | |
| **LV ejection fraction (%)** | 0.931 (0.897, 0.967) | 0.939 (0.902, 0.978) | <0.01 |
| Received Transplant |  | 0.305 (0.066, 1.405) | 0.13 |
| Age (years) |  | 1.028 (0.989, 1.068) | 0.16 |
| Female |  | 1.094 (0.350, 3.420) | 0.88 |
| Diabetes |  | 0.383 (0.157, 0.932) | 0.03 |
| Time on Dialysis (years) |  | 0.945 (0.780, 1.144) | 0.56 |

Supplementary table S2: Relationship between CMR measures and cardiovascular mortality in adjusted and unadjusted models.

| **Variable** | **Observations** | **Mean** | **95% confidence interval** |
| --- | --- | --- | --- |
| Global Native T1 (ms) | 1,000 | 1.010 | (1.010, 1.011) |
| Received Transplant | 1,000 | 2.556 | (0.572, 4.541) |
| Age (years) | 1,000 | 1.059 | (1.057, 1.060) |
| Received Transplant *Age | 1,000 | 2832730 | (-1770965, 7436425) |
| Female | 1,000 | 1.098 | (1.057, 1.139) |
| Diabetes | 1,000 | 0.761 | (0.740, 0.782) |
| Time on dialysis (years) | 1,000 | 0.886 | (0.881, 0.891) |

Table S3: Bootstrapped analysis model for global native T1. The reference category for Received transplant is ‘no’ and Diabetes is ‘yes’.

| **Variable** | **Observations** | **Mean** | **95% CI** |
| --- | --- | --- | --- |
| Left ventricular mass index (g/m^2^) | 1,000 | 1.024 | (1.023, 1.024) |
| Received Transplant | 1,000 | 392.018 | (-335.584, 1119.621) |
| Age (years) | 1,000 | 1.071 | (1.069, 1.072) |
| Received Transplant * Age | 1,000 | 2175327 | (-949747.1, 5300402) |
| Female | 1,000 | 1.426 | (1.376, 1.475) |
| Diabetes | 1,000 | 0.753 | (0.733, 0.772) |
| Time on dialysis (years) | 1,000 | 0.873 | (0.867, 0.878) |

Table S4: Bootstrapped analysis model for left ventricular mass index. The reference category for Received transplant is ‘no’ and Diabetes is ‘yes’.

| **Variable** | **Observations** | **Mean** | **95% CI** |
| --- | --- | --- | --- |
| Left ventricular ejection fraction (%) | 1,000 | 0.970 | (0.969, 0.971) |
| Received Transplant | 1,000 | 24.813 | (-10.404, 60.030) |
| Age (years) | 1,000 | 1.056 | (1.055, 1.058) |
| Received Transplant * Age | 1,000 | 2348663 | (-1163970, 5861295) |
| Female | 1,000 | 1.252 | (1.211, 1.292) |
| Diabetes | 1,000 | 0.758 | (0.738, 0.777) |
| Time on dialysis (years) | 1,000 | 0.871 | (0.866, 0.876) |

Table S5: Bootstrapped analysis model for left ventricular ejection fraction. The reference category for Received transplant is ‘no’ and Diabetes is ‘yes’.
